# The mediating role of early maladaptive schemas on the relationship between illness perception and pain coping strategies among adolescents diagnosed with migraine

**DOI:** 10.3389/fneur.2023.1128965

**Published:** 2023-03-28

**Authors:** Ozan Kayar, İlkiz Altinoğlu Dikmeer, Gülen Güler Aksu, Fevziye Toros, Aynur Özge

**Affiliations:** ^1^Department of Psychology, Çankırı Karatekin University, Çankırı, Türkiye; ^2^Department of Child and Adolescent Psychiatry, Mersin University, Mersin, Türkiye; ^3^Department of Neurology, Mersin University, Mersin, Türkiye

**Keywords:** adolescent, early maladaptive schemas, illness perception, migraine, pain coping strategies

## Abstract

**Objective:**

This study aimed to examine the mediating role of early maladaptive schemas on the relationship between illness-related perceptions and pain coping strategies among adolescents diagnosed with migraine.

**Material and methods:**

A total of 134 adolescents (aged 12–18 years) diagnosed with migraine with and without aura participated in the study. The Illness Perception Questionnaire, the Pain Coping Questionnaire, and the Early Maladaptive Schema Questionnaires Set for Children and Adolescents were used.

**Results:**

The intensity of using desperate ways of coping with pain was higher among adolescents who perceive migraine as a chronic disease (*β* = 0.199, *p* < 0.05) even if they have episodic attacks and who have higher levels of coherency in understanding the illness (*β* = 0.256, *p* < 0.01). First, full mediations of over-vigilance/inhibition and impaired autonomy/performance schema domains on these relations were observed. Second, the increases in negative cognitive (*β* = 0.199, *p* < 0.05) and emotional (*β* = 0.280, *p* < 0.01) representations related to the consequences of the illness lead to an increase in the uncontrolled and frequent use of analgesic drugs where the partial mediating role of over-vigilance/inhibition schema domain on this correlation is observed. The perceptions about the negative as well as serious consequences of migraine are related to both the self-active behaviors (*β* = 0.181, *p* < 0.05) and the conscious cognitive attempts (*β* = 0.207, *p* < 0.05) as effective coping strategies, which is an unexpected finding. The disconnection/rejection schema domain had a full mediation role on both relations.

**Conclusion:**

The results suggest that early maladaptive schemas are essential factors that affect the migraine coping processes of adolescents.

## 1. Introduction

The highest prevalence of migraine is observed during adolescence as discussed in earlier studies (1–3). Migraine is not only physical pain but also is one of the common causes that affects the family, school, and social lives of adolescents (4–6). If the migraine has a chronic course, it has been reported that the functionality of the adolescents in the abovementioned areas is more disrupted, and comorbid psychiatric disorders are encountered much more frequently (7, 8).

Recently, understanding and responding to the biopsychosocial problems caused by medical diseases such as migraine has been a special concern of health psychologists (9–11). A widely accepted approach to this area of disease is the Illness Representations Model (IRM) of Leventhal et al. (12, 13). According to this model, people develop cognitive and emotional representations of illnesses to make sense of the health threat (such as any symptom or any diagnosis) where these representations form a basis for the perception of the illness. Furthermore, it is accepted that the increase in the number of such representations is related to developing negative perspectives on illnesses. Moreover, IRM also provides a theoretical frame to comprehend the development of headache coping behaviors against illnesses-induced stress such as migraine (9). Studies showed that adolescents use dysfunctional coping strategies against migraine more often, and those dysfunctional strategies as well as the increasing frequency of the pain are related to symptoms of anxiety and/or depression and impaired functionality (14–16).

Another model to indicate the psychological aspect of pain is the Schema Therapy Model (STM) developed by Young (17), which is a promising model to understand not only the psychological disorders but also the physical problems that may cause many psychological problems through psychosocial and developmental points of view and treat them with a holistic approach (18). Recently, many studies have explored the role of early maladaptive schemas on migraine, tension headaches, breast cancer, inflammatory bowel disease, etc. (19–21). According to the STM, basic emotional needs that are not appropriately satisfied during early life and repetitive adverse experience during childhood and adolescence form early maladaptive schema domains (EMSDs) in time. These maladaptive schemas commonly include negative memories, feelings, cognitions, and bodily sensations related to the people themselves, toward others, and their life experiences (18, 22).

Despite the importance of effective ways of coping with migraine from an early age, as stated by the studies (7, 8), to the best of our knowledge, no studies focusing on the relationship between illness perception and coping with pain among adolescents exist. Therefore, exploring the common factors of IRM and STM is essential to enlighten the complexity of adolescent migraine. First, based on IRM, it is possible to state that the process of forming representations of migraine and using pain coping strategies emerges around the same time as the adolescent is diagnosed with migraine (12). Studies showed that migraine attacks emerge mostly after a stressful experience and in the early stages of life. A total of 25% of the attacks start before 10 years of age and 55% start between 10 and 20 years of age (18, 20). On the contrary, EMSDs defined by Young are related not only to migraine but also to all other early repetitive adverse experiences. In addition, it may develop during early childhood (most probably before migraine diagnosis) and negatively affect the course of illness and daily chores (17). Second, as proposed by IRM, the perception of the illness is likely to contain mostly negative cognitive and emotional representations as well as effective and ineffective cognitive, emotional, and behavioral strategies as coping mechanisms against the illness (12, 13). On the contrary, the EMSDs proposed by STM hold deep psychological constructs such as emotions, thoughts and beliefs about self and other people, and adverse experiences that might include migraine. The STM also puts forward coping strategies against the EMSDs based on negative beliefs (22). In addition, the overlapping components of these two models, both EMSDs and related coping strategies, are considered to be persistent and resistant to change. Therefore, these schemas may strongly affect the formation of the negative representations of illness (here, migraine), the pain coping strategies, and the relationships between them. In this respect, it can be inferred that these representations are relatively more flexible and open to change during adolescence.

Based on the arguments and theoretical frame discussed above, it is important to consider, in the development of migraine during the episodic phase, the role that not only the physiological aspect plays but also the cognitive, emotional, and behavioral aspects play in migraine. In this context, the present study aimed to examine the mediating role of early maladaptive schema domains on the relationship between illness-related perceptual processes and pain coping strategies among adolescents having a diagnosis of migraine with and without aura. Based on the model to be tested (refer to Supplementary Figure 1), the hypotheses of the study are as follows:

*H1. There is a significant positive relationship between negative representations related to illness perception and ineffective coping strategies with migraine, and EMSDs have a mediating role in this relationship*.

*h1. EMSDs have a mediating role in the positive relationship between negative representations and desperate (helplessness) coping strategies*.
*h2. EMSDs have a mediating role in the positive relationship between negative representations and ineffective medical remedies*.

*H2. There is a significant negative relationship between negative representations related to illness perception and effective coping strategies with migraine and EMSDs have a mediating role in this relationship*.

*h3. EMSDs have a mediating role in the negative relationship between negative representations and self-managed behavioral coping strategies*.
*h4. EMSDs have a mediating role in the negative relationship between negative representations and conscious cognitive coping strategies*.

## 2. Materials and methods

### 2.1. Participants

In this study, a power analysis for the regression factor with five predictors was conducted in G^*^Power to determine an acceptable sample size using an α level of 0.05, a power of 0.95, and a medium effect size (23). Based on these assumptions, for this study, a sufficient sample size was chosen to be 120. The primary sample of the study included 185 adolescents aged between 12 and 18 years who presented to Child and Adolescent Headache Outpatient Clinic, Mersin University Medical School (in Mersin, Turkey) with a complaint of headache. All adolescents were evaluated based on The International Classification of Headache Disorders, Third Edition (ICHD-3) diagnostic criteria by a headache specialist (AO). After the neurological assessment, 42 patients with other headache types (chronic migraine, tension-type headache, secondary headaches, etc.) were excluded. Seven adolescents were excluded due to missing information on the scales after the initial analysis of the dataset. In addition, two adolescents were excluded from the sample because their parents did not give their consent for their participation in the study. A total of 134 patients met the inclusion criteria of the study. The inclusion criteria were as follows: (1) consent of the adolescents and approval of their parents; (2) adolescents who were not having a major head injury and post-traumatic stress disorder in the last month; (3) adolescents who were not having a known medical condition that may cause headache; (4) adolescents who were not having any drug or substance misuse or abuse that might cause any headache; and (5) adolescents who were not being pregnant (for the adolescents).

### 2.2. Instruments

#### 2.2.1. Headache Questionnaire Form

In the study, neurological assessments of adolescents were carried out through a questionnaire developed by Özge et al. (24) considering the ICHD-3 diagnostic criteria. Through the questionnaire, the characteristics related to the headache of adolescents (age of onset of pain, pain frequency per month, pain intensity using a visual analog scale (VAS), etc.), the number of analgesic drugs they used per month, and the presence of any headache diagnosis among their parents, and the sociodemographic information were questioned.

#### 2.2.2. The Illness Perception Questionnaire

The Illness Perception Questionnaire (IPQ) is a scale developed by Weinman et al. (25) based on the IRM of Leventhal et al. (12, 13) to determine the perception of illness and general tendencies and feelings about the outcome of the illness of people having the most common organic illnesses. The revised form developed by Moss-Morris et al. (26) is adapted into Turkish culture by Kocaman et al. (27). The scale has three sections: type of illness, illness perception, and causes of illness. In this study, only the “illness perception” form of the scale was applied to the adolescents, considering its convenience for the purpose of the study. The illness perception form has seven subdimensions and 38 items that measure the individual's cognitive and emotional representations of their current illness that are rated by the individual on a five-point Likert-type scale. The subdimensions in the form are indicated as “timeline (acute/chronic), timeline cyclical, consequences, emotional representations, personal control, treatment control, and illness coherence.” The scale showed adequate levels of internal consistency for each subdimension, and Cronbach's α of the subdimensions were 0.88, 0.75, 0.76, 0.77, 0.75, 0.78, and 0.77, respectively. The high scores obtained from the first four subdimensions are associated with the negative representations of the illness and the high scores obtained from the other two (personal and treatment control) subdimensions are associated with the positive representations of the illness. Moreover, some researchers nominate that the higher scores of the illness coherence, the last subdimension of the scale, might be related to both negative and positive representations according to the literature of the information gathered about the illness (27, 28). Considering these explanations, this subscale is included in the analyses of our research with the assumption that it has negative representations.

#### 2.2.3. Pain Coping Questionnaire

The Pain Coping Questionnaire (PCQ) was developed by Kleinke (29) to evaluate the patterns adopted by patients with a variety of symptoms to cope with organic or psychogenic pain. The Turkish form, adapted by Karaca et al. (30), has 29 items rated on a 4-point Likert-type scale. It consists of four subdimensions (self-management, helplessness, conscious coping attempts, and medical remedies) covering both functional and non-functional pain coping strategies. The subdimensions that evaluate strategies for coping with pain using effective ways are self-active behavioral pathways to cope with pain (self-management) such as using relaxation techniques and dealing with pain through conscious cognitive attempts such as diverting attention and reinterpreting pain sensations. The other subdimensions of the scale involving disparate ways (helplessness) and seeking ineffective medical remedies measure the frequency of individuals opting for these ineffective ways. The internal consistency of all sub-dimensions was acceptable and the Cronbach's α of these subdimensions were 0.80, 0.75, 0.76, and 0.77, respectively. In the scale, the scores obtained from the relevant items for each sub-dimension are summed, and the higher scores indicate higher levels of strategies.

#### 2.2.4. Early Maladaptive Schema Questionnaires Set for Children and Adolescents

The scale was developed by Güner (31) for the Turkish culture to determine the EMSDs of children and adolescents based on the STM developed by Young (17). The self-report 97-item scale is a 5-point Likert-type scale and evaluates 15 schema dimensions and five EMSDs. According to the model, schemas were formed as a result of not meeting the basic needs at the early age, and EMSDs (disconnection/rejection, impaired autonomy/performance, impaired limits, other-directedness, and over-vigilance/inhibition) were formed through the interaction of these schema dimensions. The internal consistency of the subdimensions measuring EMSDs proved to be good in this sample, and Cronbach's α values were 0.92, 0.84, 0.86, 0.82, and 0.85, respectively. The higher scores indicated higher levels of EMSDs.

### 2.3. Procedure

Ethical approval was provided by the Social and Human Sciences Ethics Committee (protocol code 018 and date of approval 04.02.2019) of Mersin University (in Mersin, Turkey) following the Declaration of Helsinki. Data were collected between December 2018 and October 2019. In the data collection process, adolescents who applied/were referred to the Child and Adolescent Headache Outpatient Clinic, Mersin University Medical School (in Mersin, Turkey) with their parents were examined by a headache specialist (AO) and by a psychiatrist (GGA) at the Child Psychiatry Outpatient Clinic. During the neurological assessment process, the differential clinical features of the headaches and the diagnoses were determined by the specialist, and the patient's compliance with the inclusion criteria of the study was questioned. Following the procedure, the aim of the study was explained to the adolescents and to their parents who were eligible to participate in the study. The parent–child pairs willing to participate in the study signed the informed consent forms. The adolescents were asked to complete all questionnaires in paper form independently in the waiting room of the outpatient clinic. To fill the entire questionnaire, the estimated time was around 20–30 min.

### 2.4. Statistical analysis

Data analyses of the study were performed with IBM Statistical Package for Social Sciences (SPSS), version 20.0. For determining descriptive statistics, frequencies and percentages were used for categorical variables and standard deviation and mean values were used for continuous variables. Prior to the analysis, missing data were removed, and valid percentages were recorded. The Pearson correlation coefficient was used for the relationships between the variables. The significance level was accepted as at least 0.05 and 0.01.

The regression steps suggested by Baron and Kenny (32) for mediation analyses were followed to determine the indirect effects of EMSDs (mediator variable) of adolescents on the relationships between negative representations related to migraine perception (independent variable) and pain coping strategies (dependent variable). Accordingly, for a variable to be a mediator, the relationships between (a) the independent and dependent variables; (b) the independent and mediating variables; and (c) the mediating and dependent variables are expected to be significant. Finally, (d) the significant relationship between the independent and dependent variables loses its significance (full mediation) or the previous level of significance decreases (partial mediation) when the mediating and independent variables are embedded simultaneously into the regression analysis. In addition, the significance of the indirect effect is tested *via* the Sobel test (33) in Baron and Kenny's study.

## 3. Results

### 3.1. Sociodemographic and clinical characteristics of the sample

The findings on sociodemographic variables of the sample revealed that the mean of the adolescents' age was 15.41 (±1.88). Of the total number of participants, 66.4% (*n* = 89) of adolescents were girls and 33.6% (*n* = 45) were boys. As a result of the neurological assessment, 34.3% (*n* = 46) of the adolescents were diagnosed with migraine without aura and 65.7% (*n* = 88) with migraine with aura. The adolescents were also examined at the child psychiatry outpatient clinic in terms of the comorbidity of any mental disorders. According to the results, 35.1% (*n* = 47) of the adolescents diagnosed with migraine had an internalizing disorder (e.g., major depressive disorder, and anxiety disorders), 11.2% (*n* = 15) had an externalizing disorder (e.g., attention deficit hyperactivity disorder and oppositional defiant disorder), and 6.7% (*n* = 9) of them had both internalizing and externalizing symptoms. A total of 47% (*n* = 63) of the adolescents had no mental disorders. All descriptive findings regarding clinical features of the headaches as well as other sociodemographic variables of the sample are shown in Table 1.

**Table 1:** Descriptive statistics for sociodemographic and clinical characteristics of the sample (*N* = 134).

	**Response**	***n***	**%**
**Sociodemographic characteristics**			
Gender	Female	89	66.40
	Male	45	33.60
Socioeconomic level	Poor	33	24.6
	Medium	80	59.7
	Good	21	15.7
Maternal educational background	Elementary school	67	50.0
	High school	40	29.9
	Higher education (2 years)	20	14.9
	University or above	7	5.2
Paternal educational background	Elementary school	68	50.7
	High school	38	28.4
	Higher education (2 years)	19	14.2
	University or above	9	6.7
Parental condition	Together	116	86.6
	Divorced/separated	18	13.4
**Clinical characteristics**			
Neurological diagnosis (adolescent)	Migraine with aura	46	34.3
	Migraine without aura	88	65.70
Neurological diagnosis (parent)	Migraine with aura	24	40.0
	Migraine without aura	30	50.0
	Chronic migraine	6	10.0
Mental disorder diagnosis	Internalization disorders	47	35.1
	Externalization disorders	15	11.2
	Comorbidity	9	6.7
	No diagnosis	63	47.0
Analgesic drug use (per month)	10 and more	21	15.7
	10 and less	86	64.2
	No using	27	20.1
		**Mean**	**SD**
Age (years)		15.2	1.88
Age of onset of pain (range 3–17 years)		12.48	2.63
Pain frequency (per month) (range 2–15)		9.88	3.76
Pain intensity (range 4–10)		7.70	1.24

### 3.2. Correlations among negative representations related to illness perception, pain coping strategies, and early maladaptive schema domains

The analyses revealed significant correlations among the negative representations related to illness perception, effective and ineffective coping strategies for migraine, and EMSDs of the participants (refer to Supplementary Table 1).

Significant relationships between some negative representations related to the illness perception and pain coping strategies of the adolescents were detected. According to these results, which are in line with our hypothesis H1, all subdimensions of the IPQ measuring negative representations of illness perception were positively correlated with the scores of coping with pain in desperate ways (ranging from 0.199 to 0.460). The scores of coping with pain by seeking ineffective medical remedies—the other ineffective pain coping strategy—correlated significantly and positively with the adolescents' perception of the consequences of the illness (*r* = 0.199, *p* < 0.05) and emotional representations related to the illness (*r* = 0.280, *p* < 0.01) scores only. On the one hand, contrary to our expectations in our hypothesis H2, there was a positive and significant relationship between some of the negative representations related to the illness perception and strategies for coping with migraine in effective ways. According to these results, the scores of adolescents' perception of the consequences of the illness were positively correlated with the scores of handling pain through conscious cognitive attempts (*r* = 0.207, *p* < 0.05) and using self-active behavioral pathways (*r* = 0.181, *p* < 0.05). Besides, coping with pain through scores of conscious cognitive attempts were positively correlated with the scores of the timeline acute/chronic subdimension (*r* = 0.224, *p* < 0.01) and the timeline cyclical subdimension (*r* = 0.218, *p* < 0.05) of the illness perception questionnaire.

On the other hand, scores for negative representations related to illness perception were correlated with many EMSDs. According to these results, the perception of the consequences of the illness scores of adolescents was moderately and positively correlated with all subdimensions of the early maladaptive schema questionnaires set for children and adolescents (EMSQSCA) (ranging from 0.315 to 0.469). Four subdimensions of the EMSQSCA, other than the impaired limits domain, were positively correlated with the perceptions of the duration of the illness (cyclical) scores (ranging from 0.172 to 0.235), and the emotional representations of the illness scores (ranging from 0.188 to 0.457). As the adolescents' coherency in understanding illness scores increased, so did their scores in the schema domains of disconnection/rejection (*r* = 0.180, *p* < 0.05), impaired autonomy/performance (*r* = 0.350, *p* < 0.01), and over-vigilance/inhibition (*r* = 0.298, *p* < 0.01). Perception of the duration of the illness (acute/chronic) scores of the participants was only positively correlated with the over-vigilance/inhibition domain (*r* = 0.178, *p* < 0.05).

Moreover, analyses to determine the relationships between adolescents' EMSDs and strategies for coping with pain using ineffective ways also showed some significant correlations. According to these results, as the scores of all maladaptive schema domains in the EMSQSCA increased, the scores of coping with pain in desperate ways also increased (ranging from 0.189 to 0.412). Besides, there were positive correlations between coping with pain by seeking ineffective medical remedies scores and disconnection/rejection (*r* = 0.267, *p* < 0.01) and over-vigilance/inhibition (*r* = 0.292, *p* < 0.01) score only. However, there were also significant relationships between some EMSDs and strategies for coping with migraine effectively. According to these results, the impaired limits domain scores were negatively (*r* = −0.192, *p* < 0.05), and disconnection/rejection domain scores were positively (*r* = 0.178, *p* < 0.05), correlated with the scores of using the self-active behavioral pathways to cope with pain. Similarly, there were significant positive correlations among the scores of coping with pain through conscious cognitive attempts, the disconnection and rejection domain (*r* = 0.228, *p* < 0.01), and the impaired autonomy and performance domain (*r* = 0.177, *p* < 0.05). These bivariate correlations suggest that the following analyses of mediation can be performed.

### 3.3. Mediating effects of early maladaptive schema domains in the relationship between negative representations related to illness perception and ineffective coping strategies for migraine

In this study, regarding our first subhypothesis (h1), a series of regression analyses were carried out considering the mediator role of EMSDs between negative representations related to the illness perception and coping with pain in desperate ways—one of the ineffective pain coping strategies. Our results showed that there were three full mediations in the abovementioned relations.

First, following Baron and Kenny's (32) regression steps (a, b, and c) it was convenient to question the potential mediation of the over-vigilance/inhibition schema domain on the relationship between perception of the duration of the illness (acute/chronic) and coping with pain in desperate ways. In the last regression step carried out, the significant relationship between the perception of the duration of the illness (acute/chronic) and coping with pain in desperate ways (*β* = 0.199, *p* < 0.05) disappeared when the over-vigilance/inhibition schema domain was added to the model (*β* = 0.129, *p* > 0.05). Sobel test results indicated a significant indirect effect (*z* = 1.93, *p* = 0.054); therefore, over-vigilance/inhibition schema domain was accepted as a full mediator variable (refer to Supplementary Figure 2). Following the same procedure, second and third mediations were found to link the coherency in understanding the illness and coping with pain in desperate ways (refer to Supplementary Figure 3). For the second mediation, it was observed that the significant relationship between coherency in understanding illness and coping with pain in desperate ways (*β* = 0.256, *p* < 0.01) disappeared with the addition of the impaired autonomy/performance schema domain to the model (*β* = 0.149, *p* > 0.01). The Sobel test results confirmed the full mediator role of impaired autonomy/performance schema domain in this interaction (*z* = 3.08, *p* = 0.002). Similarly, the significant relationship between coherency in understanding illness and coping with pain in desperate ways (*β* = 0.256, *p* < 0.01) vanished by adding the over-vigilance/inhibition schema domain to the model (*β* = 0.146, *p* > 0.01). Sobel test displayed a significant indirect effect indicating the full mediation of the over-vigilance/inhibition schema domain (*z* = 2.94, *p* = 0.003).

Coping with pain by seeking ineffective medical remedies is the other ineffective coping strategy that was mentioned in our subhypothesis h2. A series of regression analyses were carried out to assess the mediator role of EMSDs between negative representations related to illness perception and coping with pain by seeking ineffective medical remedies. Following the Baron and Kenny (32) procedure, the potential mediation role of the over-vigilance/inhibition schema domain was questionable in terms of the relationship between the two sub-dimensions of IPQ and coping with pain by seeking ineffective medical remedies. The results showed both full and partial mediations. When the over-vigilance/inhibition schema domain was added to the model, the significant relationship between perception of the consequences of the illness and coping with pain by seeking ineffective medical remedies (*β* = 0.199, *p* < 0.05) receded (*β* = 0.079, *p* > 0.01). The Sobel test confirmed the indirect effect and the full mediator role of the over-vigilance/inhibition schema domain on this relationship (*z* = 3.04, *p* = 0.002) (refer to Supplementary Figure 4). For the partial mediation, it was observed that the relationship between emotional representations related to the illness and coping with pain by seeking ineffective medical remedies (*β* = 0.280, *p* < 0.01) decreased by incorporating the over-vigilance/inhibition schema domain to the model in the last regression analysis. However, the existing direct effect remained significant (*β* = 0.207, *p* > 0.01). The Sobel test showed that the indirect effect of the over-vigilance/inhibition schema domain was significant, and that domain had a partial mediator role in this relationship (*z* = 3.02, *p* = 0.002) (refer to Supplementary Figure 5).

### 3.4. Mediating effects of early maladaptive schema domains in the relationship between negative representations related to illness perception and effective coping strategies for migraine

To test the other hypotheses of the study, considering the mediator role of EMSDs in the negative relations between negative representations related to illness perception and effective behavioral (h3) and cognitive (h4) pain coping strategies, a series of regression analyses were carried out. The results obtained indicated the existence of two full mediations of the disconnection/rejection schema domain.

Contrary to our subhypothesis h3, the results revealed that the significant relationship between perception of the consequences of the illness and using self-active behavioral pathways to cope with pain (*β* = 0.181, *p* < 0.05) lost its significance when disconnection/rejection schema domain was added to the model (*β* = 0.126, *p* > 0.05). Indirect effects were found significant indicating the disconnection/rejection schema domain was a full mediator variable (*z* = 1.95, *p* = 0.051) (refer to Supplementary Figure 6). Analyses showed another mediation—similar to our aforementioned finding—which was inconsistent with our subhypothesis h4. It was observed that the significant positive relationship between perception of the consequences of the illness and coping with pain through conscious cognitive attempts (another effective pain coping strategy) (*β* = 0.207, *p* < 0.05) disappeared by adding the disconnection/ rejection schema domain to the model (*β* = 0.129, *p* > 0.05). According to the results of the Sobel test, the indirect effect was statistically significant (*z* = 2.44, *p* = 0.013), and the disconnection/rejection schema domain was considered as a full mediator variable (refer to Supplementary Figure 7).

## 4. Discussion

In this study, based on the Illness Representations Model (12, 13), it was assumed that the emotional and cognitive aspects of the negative illness perceptions of adolescents with migraine are closely associated with their coping strategies with pain. Similarly, the Schema Therapy Model (17) was applied to have a better understanding of the relations between those variables. Therefore, multiple mediation models to determine the role of EMSDs in this relation were brought forward.

Related to our subhypothesis h1, the results revealed that two EMSDs (over-vigilance/inhibition and impaired autonomy/performance) had three mediating roles on the significant positive relationships between the two components of negative representations of illness (timeline acute/chronic and coherency in understanding illness) and the ineffective coping strategy of migraine, namely, coping with pain in desperate ways.

We found that adolescents' coping with their migraine in desperate ways was related to their perception of the duration of their illness and with their coherency in understanding migraine where the over-vigilance/inhibition schema domain had a full mediator role. It is important to interpret this result comprehensively. To begin with, it is essential to consider that the coherency of illness might be perceived as both positive and negative depending on the accuracy and reliability of the information gathered about the illness (27, 28). When the results of the study are scrutinized, we observed that the scores of the coherency of understanding the illness were positively correlated with negative representations of the illness in addition to the EMSDs and desperate ways of coping with pain. This might be due to a lack of knowledge concerning migraine even though they have a considerable level of understanding of their illnesses. Possible misattributions toward their migraine might lead them to believe that pain is chronic and will never end although it is already at the episodic phase. In this context, it is possible that the adolescents might develop some maladaptive schemas linked with those negative representations, particularly pessimism and punitiveness schemas within the over-vigilance/inhibition domain. In fact, people with a pessimism schema are generally prone to be negative and the ones with the punitiveness schema usually internalize the negative aspects of their lives believing that they need to suffer (22, 34). This harsh disposition toward themselves combined with their pessimistic beliefs about life might lead the adolescents to have incorrect and negative representations of migraine and to believe they will never get well.

Considering the results of mediation analyses, we noticed that the EMSDs triggered by the negative representations of illness play an important role in embracing desperate ways of coping. “Surrender” is a schema coping style identified by Young (17), and people with pessimism schemas surrender to their schemas and tend to minimize the positive events while expecting the worst. Even if things are on track, they would believe that it is only temporary (22, 35). An example of the surrender concept for punitiveness is likely to be harsh, critical, and punitive on the others but especially on himself/herself. This is mostly about rigid misbeliefs such as “Everything should be perfect” or “This is happening because I deserve to be punished and this pain is my punishment” (22, 34). It is possible that the over-vigilance/inhibition schema domain, which includes the pessimism and punitiveness schemas, and desperate coping are similar in content. Furthermore, this similarity may contribute to the mediation effect found in this study. It has been suggested that the underlying roots of coping with pain in desperate ways are related to cognitive distortions (e.g., catastrophizing, all-or-none thinking, and personalization) about the pain (29). Having those distortions, the patients may apply coping strategies that make them feel incompetent and desperate against the increasing pain. The negative feelings toward pain make it even harder to tackle this distress emotionally. However, these ineffective strategies are likely to apply and to trigger pessimism and punitiveness.

Another mediation effect was found between coherency in understanding illness and coping with pain in desperate ways, where the impaired autonomy/performance schema domain was the mediator. Consistent negative and probably incorrect attributions of adolescents toward migraine might trigger some schemas within the impaired autonomy/performance schema domain. Two of them—vulnerability to harm or illness schema and failure schema—might lead to using desperate coping strategies. The first one involves an ongoing expectancy of the catastrophe which causes an exaggerated and unrealistic fear (22). Adolescents generally tend to be more sensitive to any kind of input reinforcing their negative emotions and catastrophizing beliefs about migraine as they expect something bad to happen. This may lead to feelings of hopelessness and/or helplessness about the pain. Thus, adolescents become reluctant to seek effective strategies. Similarly, seeking desperate ways of coping might increase failure schema triggered by the distorted negative thoughts of young people about their migraine. The dominant idea of failure schema is the self-perception of being stupid, incompetent, or a loser. This self-perception of failure does not necessarily fit the reality. These people often avoid situations where they can easily succeed (34, 35). Therefore, it appears that those misbeliefs and maladaptive schemas become an obstacle to overcoming the pain.

With respect to subhypothesis h2 of this research, if the adolescents perceive their migraine as an illness causing a strong negative effect on their functionality and experience intense negative feelings such as anxiety and/or depression symptoms, it may be expected that the schemas within the over-vigilance/inhibition domain may be triggered more. Therefore, the predictor value of the negative behavioral and cognitive representations concerning the negative consequences of the illness on coping with pain by seeking ineffective medical remedies might be due to the indirect and strong effects of those schemas.

The part of coping with pain through ineffective medical remedies of the scale evaluates uncontrolled and unconscious use of analgesic drugs which do not belong to the treatment protocol of pain attacks (5). Negativity/pessimism and emotional inhibition schemas of the over-vigilance/inhibition domain and some maladaptive coping methods used to manage these schemas may have a role in ineffective coping strategies. For instance, the negativity/pessimism schema is usually related to increased anxiety due to rigid misbelief about catastrophic expectations on health, finance, and/or social lives (22). Frequent activation of this schema might make the adolescents more prone to lay on any type of coping strategy to avoid negative feelings, including uncontrolled use of analgesic drugs. Moreover, the emotional inhibition schema is characterized by the core belief that negative urges and feelings toward problems should excessively be repressed and controlled. People having this schema expect everything to be all right to have positive feelings. These high standards usually come from oppressive and intolerant family attitudes (22, 34). Those core beliefs combined with the wishes to be accepted by others might increase the use of uncontrolled and frequent use of analgesic drugs by adolescents as easily obtained over-the-counter pills would give temporary relief (5). Bearing in mind that frequent use of these pills might help adolescents to suppress their emotions about their illnesses, they tend to think that they have no medical problems at all. On the contrary, the studies indicate that the root cause of the analgesic drugs used by adolescents in times of pain is advice from their parents (5, 36, 37). Having this in mind, it is possible that the adolescents are communicating their pain and related suppressed negative emotions indirectly by asking for analgesic drugs from their parents (36). At the same time, asking for pills can also be a way to seek parental attention and get emotional care (5). As a matter of fact, in families where emotional inhibition is encouraged, parents often tend to ignore the negative feelings of their children, but they do not tend to empathize with the negative feelings. Therefore, adolescents may choose not to express their emotions (22, 34) while communicating through analgesic drugs might be considered as a cry for help for emotional support (5).

Our hypotheses h3 and h4 emphasize the mediating role of EMSDs in the negative relations between negative illness representations and the effective coping strategies with migraine (h3: self-active behavioral pathways; h4: conscious cognitive attempts). However, contrary to our expectations, negative representations of the adolescents were not related to any of the effective coping strategies. Surprisingly, the increase in perception of the consequences of migraine was associated with the use of effective strategies. Moreover, both relationships were fully mediated by the disconnection/rejection schema domain.

It has previously been stated that negative cognitive and emotional representations of the consequences of migraine could be related to some parental attitudes that contribute to the development of schemas within the over-vigilance/inhibition schema domain. In addition, the results showed that the disconnection/rejection schema domain, which is related to having loose family ties, is triggered by negative representations on the consequences of the illness. Thus, adolescents frequently use effective behavioral and cognitive strategies. To discuss this unexpected result, it is reasonable to analyze the core beliefs and schema coping styles related to the schemas in the disconnection/rejection domain, particularly the abandonment/instability, mistrust/abuse, and social isolation/alienation schemas. These schemas include beliefs such as: “One or more of my significant others will abandon me and I will end up alone eventually,” “I shouldn't trust anyone including my parents as they can disappoint me or betray me” or “I have no one to meet my basic emotional needs such as warmth, security, care, guidance, etc.” (22, 34). To get adapted to these schemas, people usually develop schema coping styles to prevent disappointment or getting hurt. The most frequently used ones are used to avoiding forming intimate relationships, are not used to trust even the closest people, are not used to expect the basic needs to be met, and are used to adopt an attitude of not asking anything from others (22, 35). Taking all those into consideration, it is believed that those maladaptive schemas and schema coping styles might lead adolescents to cope with the difficulties of life by themselves. However, there may be other variables contributing to the exploration and development of effective cognitive and emotional strategies.

Within families where disconnection/rejection schemas have developed, a lack of social and emotional support is frequently seen (34). Children and adolescents who experience helplessness in coping with their headaches may express their pain more often by seeking social support and empathy from others (5, 38). In a recent study, the effects of parental attitudes toward patients migraine on their adolescent children were investigated (39). Among the adolescents studied, 50% of those whose parents had chronic migraine and 27% of patients with migraine with and without aura reported that they did not receive any support when they underwent pain attacks. Parents who display catastrophizing and desperate attitudes toward their headaches tend to report higher degrees of pain (5). Some studies indicate that when parents are not good role models for their children in coping with pain, and cannot support their children, they contribute to the development of pain-oriented and functional coping strategies in adolescents with similar pain complaints (5, 40). A recent qualitative study reported that parents with ineffective pain coping strategies might lead various positive outcomes in their adolescent children such as having more empathy and learning to deal with pain more effectively despite the challenges (40). In line with the literature discussed in (5, 36), the findings indicated that most of our participants shared an environment with at least one parent suffering from headaches. It is possible to say that the parents might not sufficiently support their children during pain attacks, which in turn, might contribute to the development of the disconnection/rejection schema domain.

In addition to the abovementioned family environment and parental attitudes; personal, age-related, and developmental factors might play a role in searching for effective pain coping strategies (5, 41). In fact, researchers state that the coping strategies of adolescents change by their age, cognitive level, and experience (14, 41, 42). For instance, it is possible that the pain coping styles of adolescents may be affected by the efficacy of previously used strategies (41). Thus, the adolescents having schemas of disconnection/rejection may have failed in the past when they attempted to cope with their pain by themselves. Considering those failures, adolescents may come up with more functional and effective coping strategies through trial and error. In a study, older adolescents used more effective coping strategies as they have more experience and knowledge about which strategies would work (42). In other words, they can differentiate the strategies that work or not and explore new effective ways of dealing with pain. However, it is important to recognize the effects of cognitive development as they grow up. Starting with adolescence, individuals start to use more abstract thinking, more hypothetical reasoning, and logical generalizations for problem-solving (43). Such cognitive skills will help the adolescents to learn from previous experiences and be more open to discovering more effective strategies (5, 43). Various studies report that adolescents having different types of pain are better at controlling their emotions and drawing upon effective behavioral and cognitive strategies than younger children since they had more developed cognitive skills (5, 42). In light of the results displayed above, it is believed that adolescents are motivated to explore more effective active coping strategies to reduce their pain with the effects of schemas in the disconnection domain and with the help of their increasing cognitive skills.

## 5. Limitations

Despite the valuable findings, there are some limitations to our study. The clinic-based, single-center, and cross-sectional design of the study limits the generalization of the results. Conducting a larger study with a control group and longitudinal or follow-up studies will contribute to validating the findings. In addition, the data were gathered from the adolescents who applied to the child psychiatry clinic or who were referred to the child psychiatry clinic by the doctors of the pain unit. Therefore, it is likely that the comorbidity of the psychiatric diagnoses is high, and it is best to limit the results to a small group rather than generalize them to the normal population. The effects of psychiatric illnesses accompanying migraine on the main variables of the study were not analyzed due to the non-normal distribution of the small sample size. It can be inferred that the pain coping strategies and EMSDs of the adolescents were affected by their current psychopathologies. To differentiate these effects, it would be best to conduct studies on adolescents having migraine with or without comorbid psychopathologies.

The other limitation of the study is that the variables related to the migraine coping strategies were limited only to the four subscales of the PCQ. Moreover, the relations between the main variables of our study were analyzed in terms of the mediator roles of EMSDs rather than the maladaptive schemas that make up these EMSDs. Further studies focusing on these specific maladaptive schemas and alternative ways of pain coping adapted by adolescents would give more comprehensive results.

## 6. Contributions and suggestions

There are many studies focusing on the early onset of migraine, the psychological problems accompanying the migraine diagnosis, or the distorted functionality of the adolescents (4–6), but studies that examine pediatric migraine with the STM perspective are limited (44, 45). Our findings will contribute to the researchers who would study migraine in children and adolescents through the psychosocial and developmental perspective of STM. Therefore, we believe that our findings might provide an essential contribution to the researchers who study childhood and adolescent headaches within the context of STM and who wish to carry out their studies in this field on solid foundations. Further research on the subject will help the clinicians working with headache problems of children and adolescents in multidisciplinary clinical settings to understand the young patients and family dynamics in a holistic approach.

Based on our mediation findings, interventions against EMSDs—whose mediating effects are observed—would contribute to reducing or eliminating the negative impact of emotional and cognitive representations of the illness on the choice of coping strategies. However, both EMSDs and coping strategies that are actively used by adolescents may become an addiction for the rest of their lives (22, 46, 47). Therefore, our findings would help the practitioners to initiate guidance and migraine treatment protocols by preventing the development of early maladaptive schemas and encouraging more adaptive and functional coping strategies.

## 7. Conclusion

To the best of our knowledge, this study is the first to focus on the complex relationship between illness perception, pain coping strategies, and EMSDs in adolescents with migraine. Our results revealed that negative representations of adolescents about migraine and ineffective pain coping strategies are related to EMSDs. Additionally, our results for mediation analyses constituted a significant part of the early maladaptive schemas on using desperate ways of coping with pain and uncontrolled use of analgesic drugs as well as developing effective strategies.

## Data availability statement

The raw data supporting the conclusions of this article will be made available by the authors, without undue reservation.

## Ethics statement

Ethical approval was provided by the Mersin University Social and Human Sciences Ethics Committee (protocol code 018 and date of approval 04.02.2019), following the Declaration of Helsinki. Written informed consent to participate in this study was provided by the participants' legal guardian/next of kin.

## Author contributions

OK and AÖ designed the study. OK, GG, FT, and AÖ were involved in data collection. OK, İA, and GG analyzed the data. OK and İA wrote the original draft. İA, FT, GG, and AÖ provided a critical review of the original draft. All authors read and approved the content of the manuscript.
