# Corrigendum: The mediating role of early maladaptive schemas on the relationship between illness perception and pain coping strategies among adolescents diagnosed with migraine

**DOI:** 10.3389/fneur.2023.1209596

**Published:** 2023-05-10

**Authors:** Ozan Kayar, İlkiz Altinoğlu Dikmeer, Gülen Güler Aksu, Fevziye Toros, Aynur Özge

**Affiliations:** ^1^Department of Psychology, Çankırı Karatekin University, Çankırı, Türkiye; ^2^Department of Child and Adolescent Psychiatry, Mersin University, Mersin, Türkiye; ^3^Department of Neurology, Mersin University, Mersin, Türkiye

**Keywords:** adolescent, early maladaptive schemas, illness perception, migraine, pain coping strategies

In the published article, there was an error. The “**Acknowledgments**” statement was mistakenly not included in the publication. The correct statement appears below.

