# Supplementary material for: The mediating role of early maladaptive schemas on the relationship between illness perception and pain coping strategies among adolescents diagnosed with migraine
Source: Front Neurol. 2023 Mar 28;14:1128965. doi: 10.3389/fneur.2023.1128965 (PMC10086163; doi:10.3389/fneur.2023.1128965)
Supplement: Supplementary file 1 [file Data_Sheet_1.docx]

***Supplementary Material***

**1 Supplementary Figures**

**
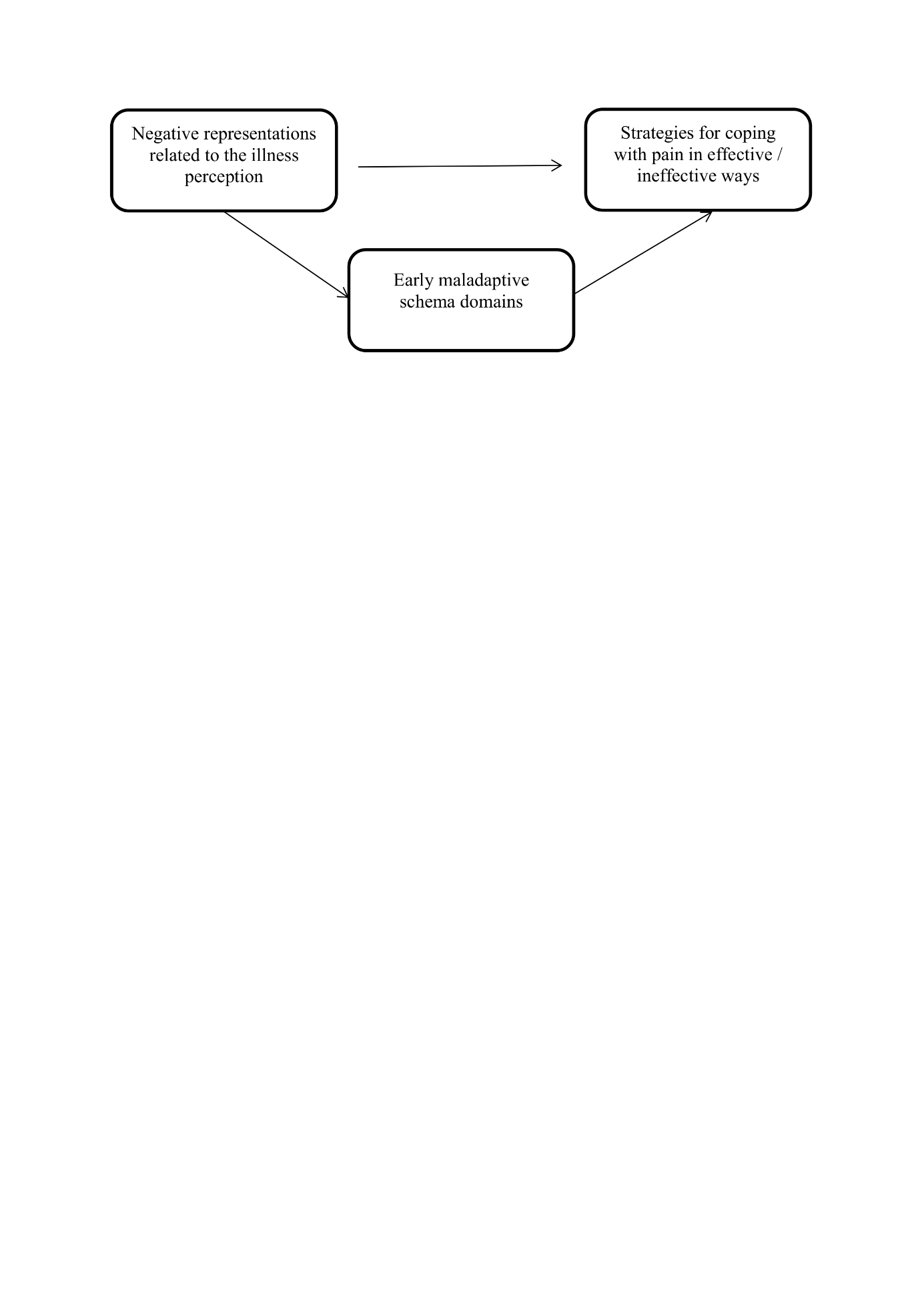
**

**Supplementary Figure 1.** The hypothesized model: early maladaptive schema domains as mediators.

*
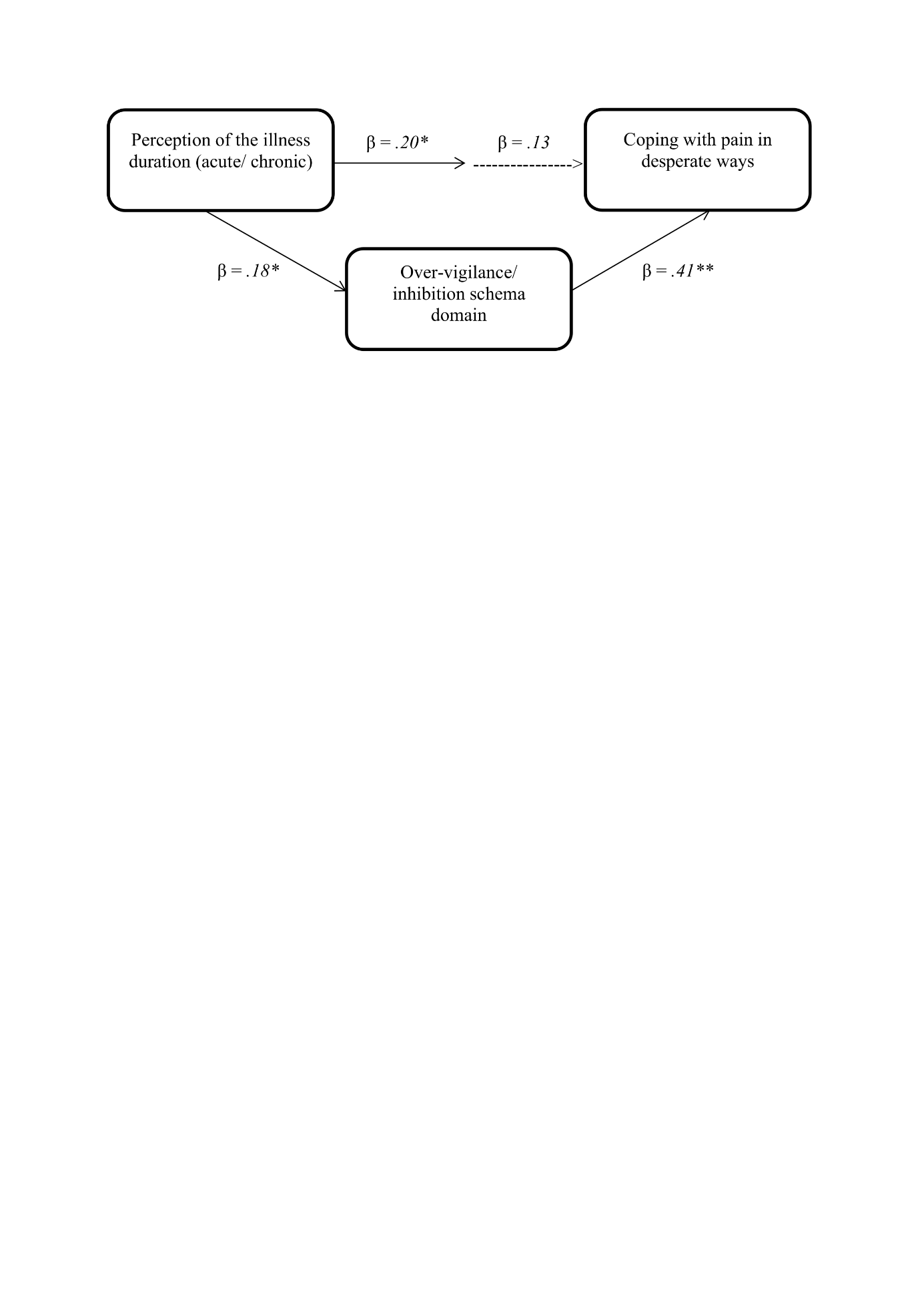
*

*Note. *p < 0.05, **p < 0.01*

**Supplementary Figure 2.** The mediating role of overvigilance/ inhibition schema domain in the correlation between perception of duration of the illness (acute/ chronic) and coping with pain in desperate ways


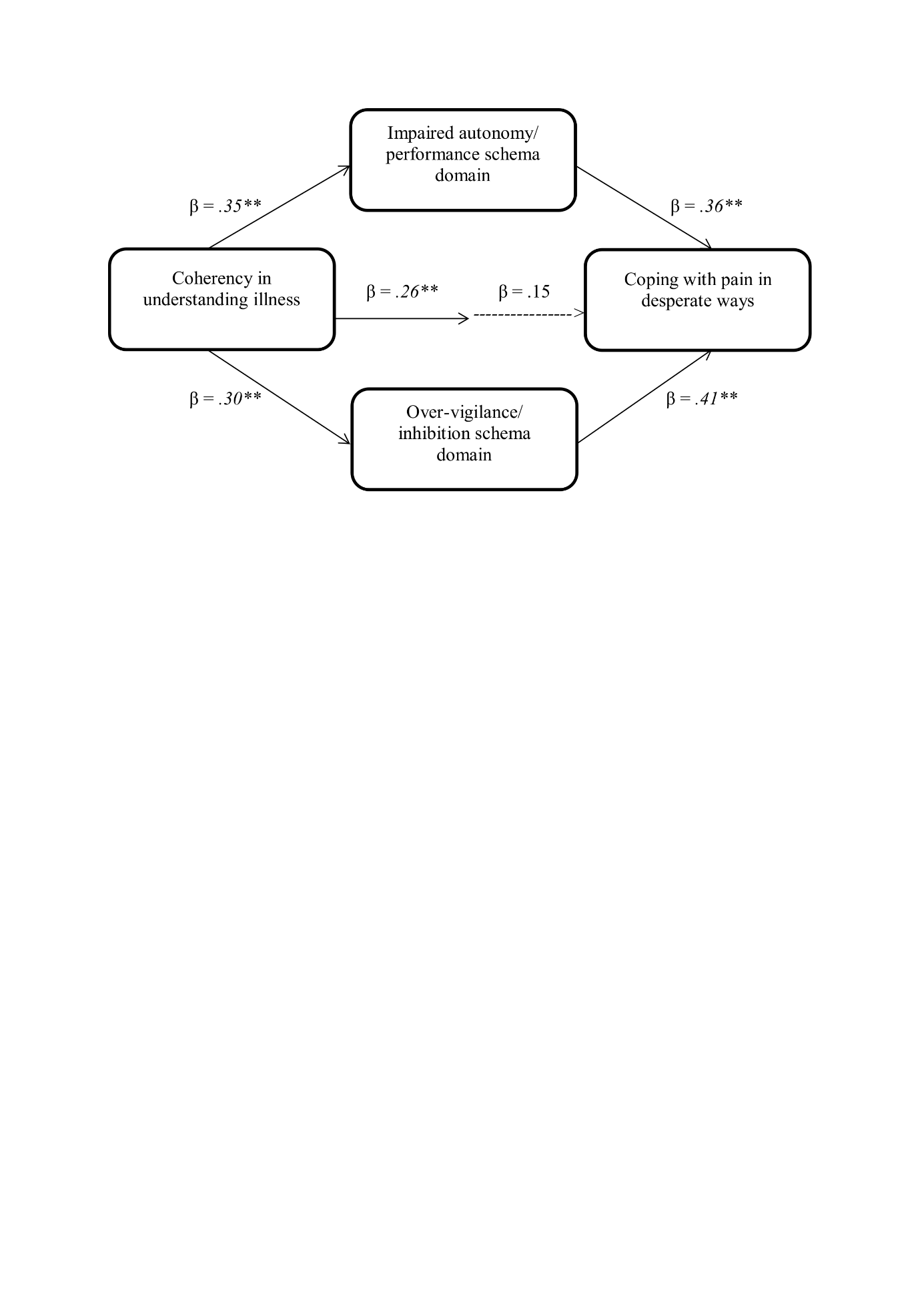


*Note. **p < 0.01*

**Supplementary Figure 3.** The mediating role of impaired autonomy/ performance and overvigilance/ inhibition schema domains in the correlation between coherency in understanding illness and coping with pain in desperate ways


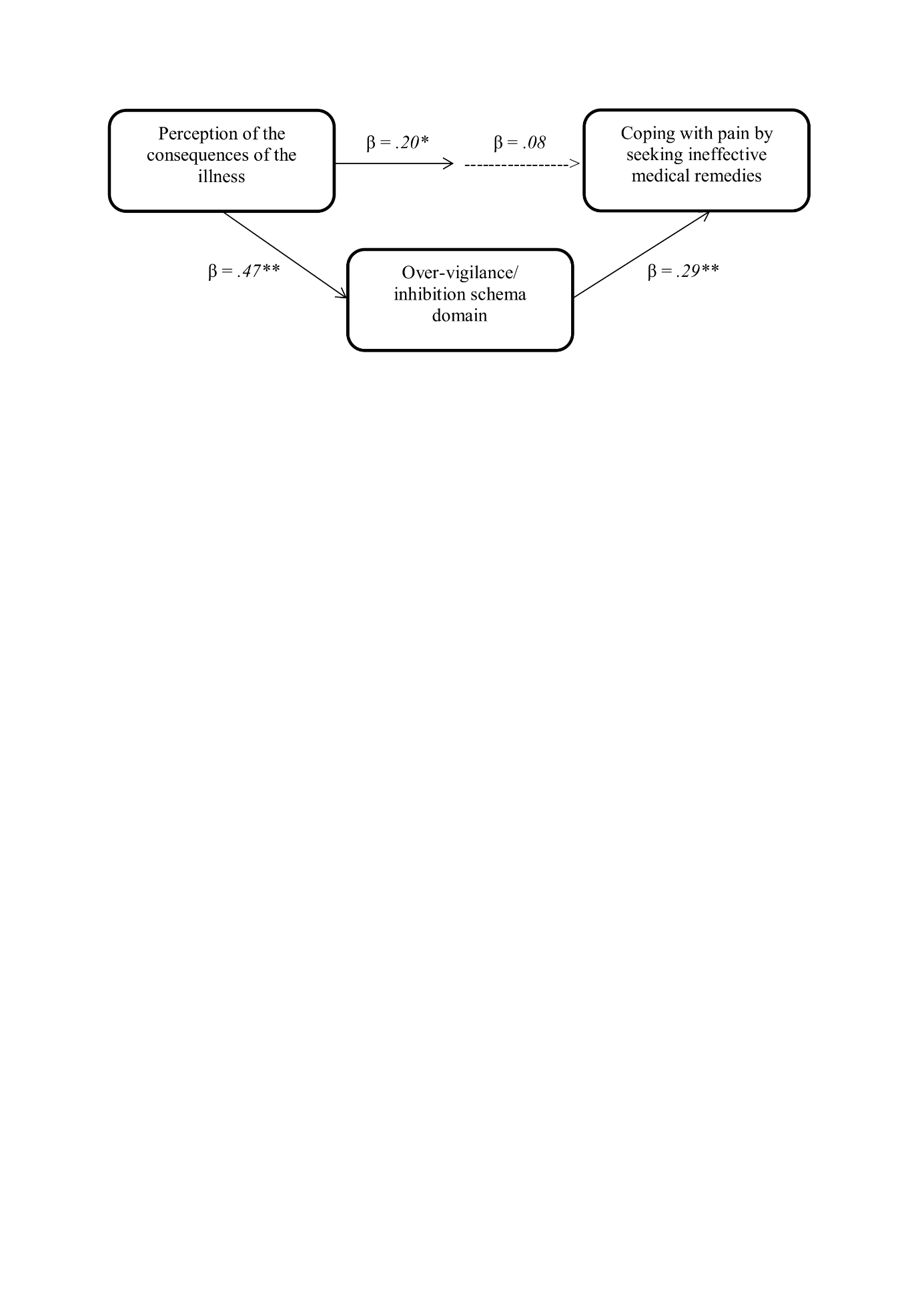


*Note. *p < 0.05, **p < 0.01*

**Supplementary Figure 4.** The mediating role of overvigilance/ inhibition schema domain in the correlation between perception of the consequences of the illness and coping with pain by seeking ineffective medical remedies


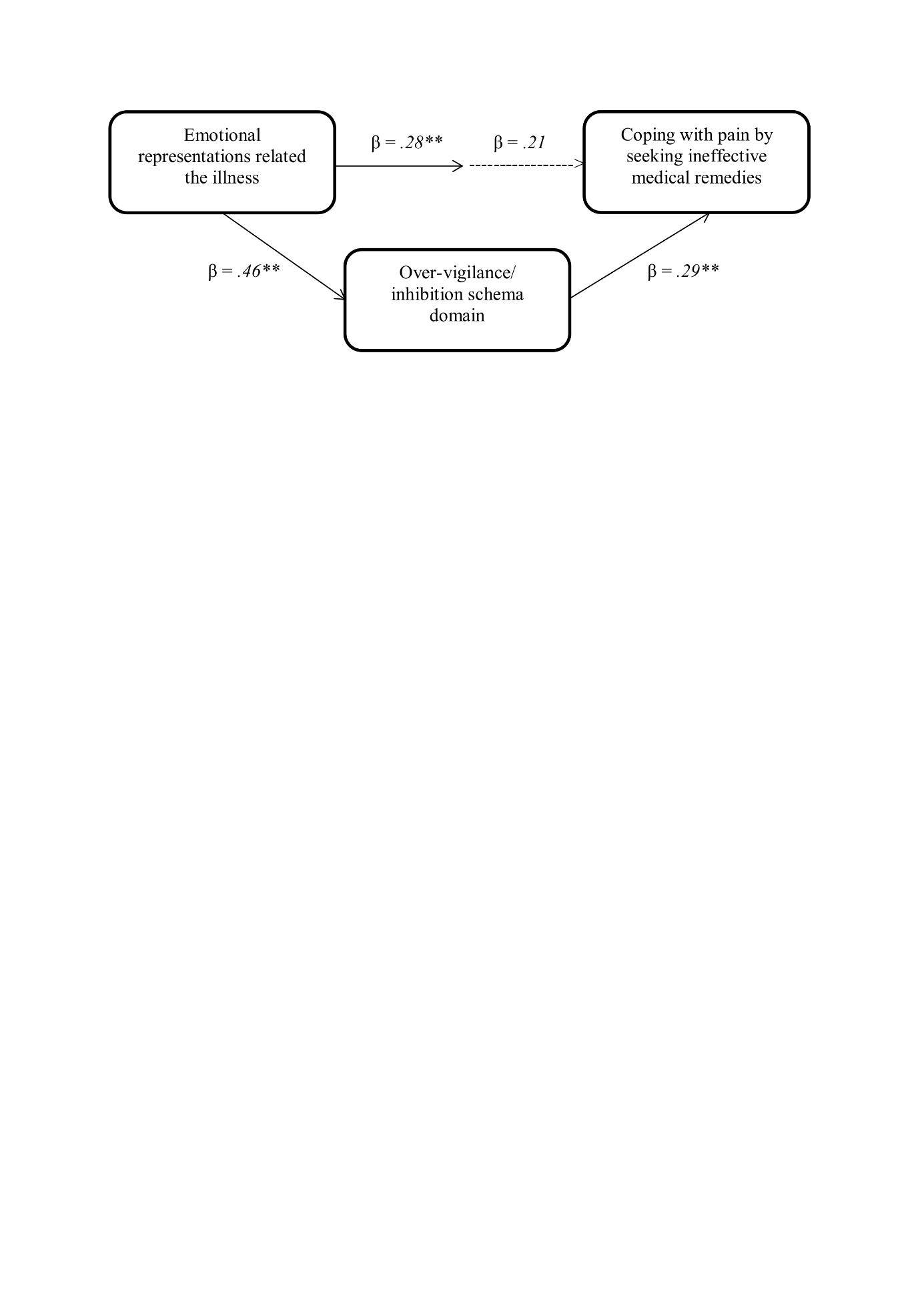


*Note. **p < 0.01*

**Supplementary Figure 5.** The mediating role of overvigilance/ inhibition schema domain in the correlation between emotional representations related the illness and coping with pain by seeking ineffective medical remedies


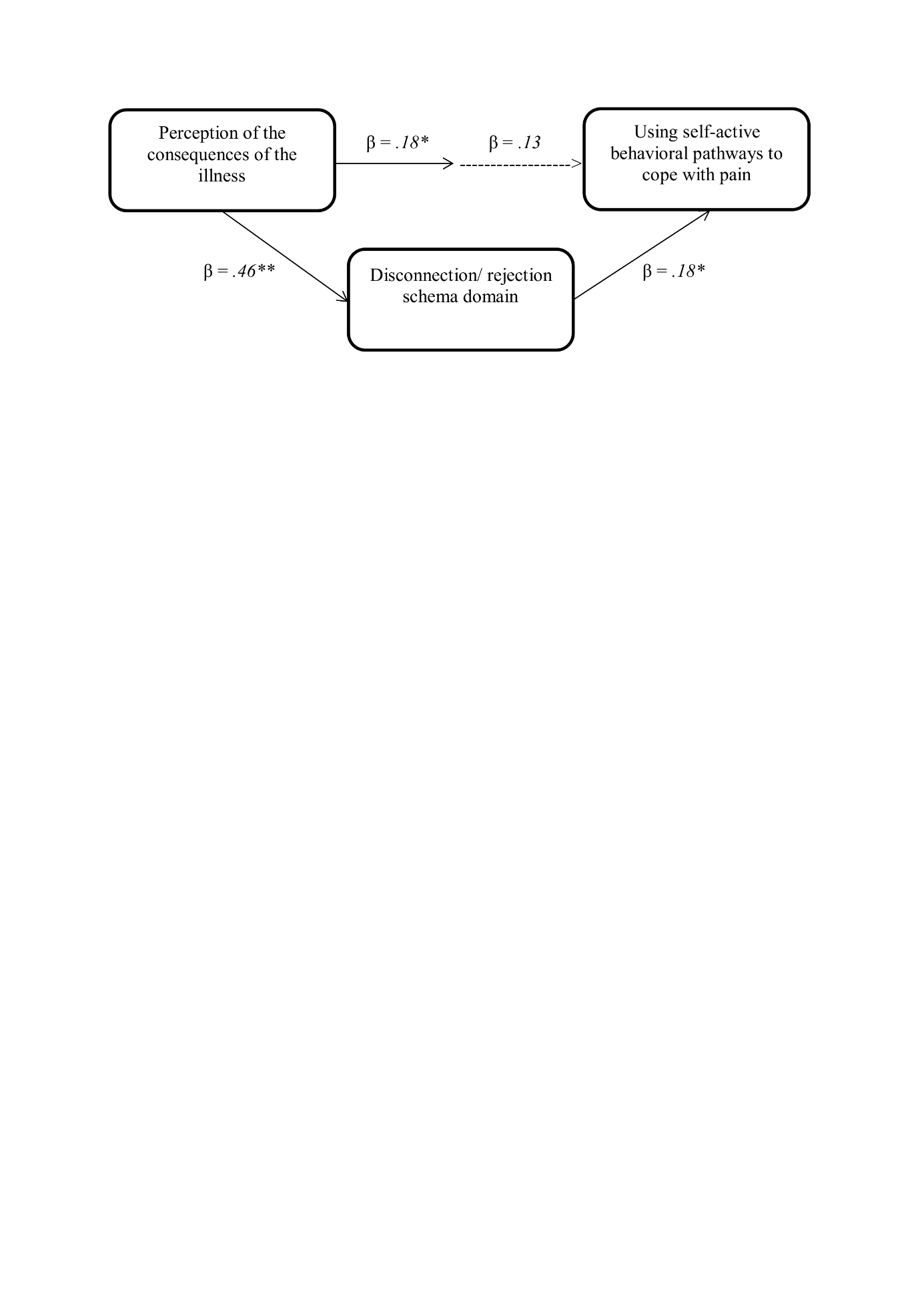


*Note. *p < 0.05, **p < 0.01*

**Supplementary Figure 6.** The mediating role of disconnection/ rejection schema domain in the correlation between perception of the consequences of the illness and using self-active behavioral pathways to cope with pain

***
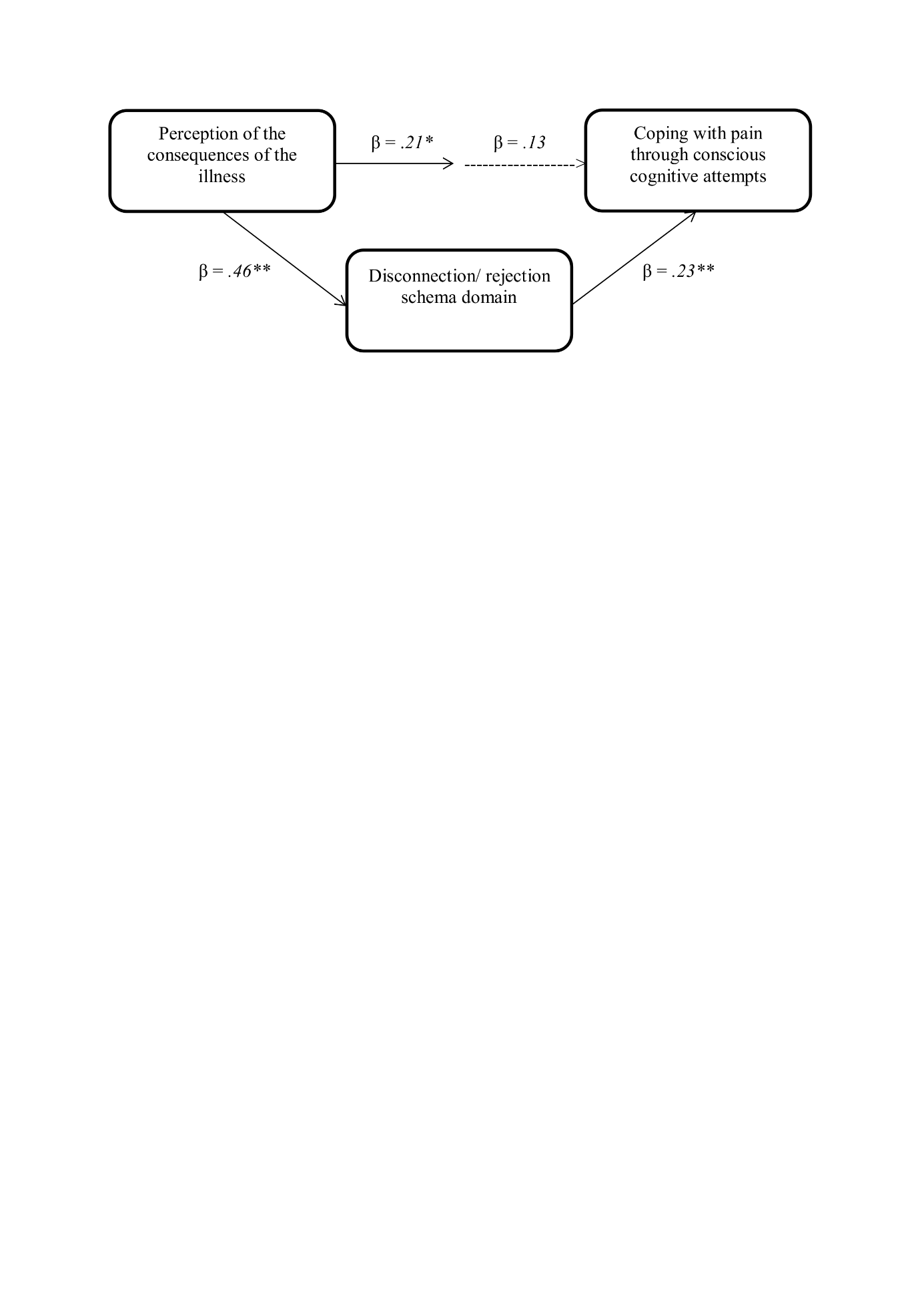
***

*Note. *p < 0.05, **p < 0.01*

**Supplementary Figure 7.** The mediating role of disconnection/ rejection schema domain in the correlation between perception of the consequences of the illness and coping with pain through conscious cognitive attempts

**2 Supplementary Tables**

**Supplementary Table 1.** Correlations among the study variables (N = 134).

|  | 1 | 2 | 3 | 4 | 5 | 6 | 7 | 8 | 9 | 10 | 11 | 12 | 13 | 14 | 15 | 16 |
| --- | --- | --- | --- | --- | --- | --- | --- | --- | --- | --- | --- | --- | --- | --- | --- | --- |
| **1. PDI (A/C)** | 1 |  |  |  |  |  |  |  |  |  |  |  |  |  |  |  |
| **2. PCI** | **0.172** | 1 |  |  |  |  |  |  |  |  |  |  |  |  |  |  |
| **3. PPC** | 0.074 | ***0.259*** | 1 |  |  |  |  |  |  |  |  |  |  |  |  |  |
| **4. PTC** | 0.061 | 0.155 | ***0.280*** | 1 |  |  |  |  |  |  |  |  |  |  |  |  |
| **5. CUI** | **0.174** | ***0.380*** | **0.210** | 0.127 | 1 |  |  |  |  |  |  |  |  |  |  |  |
| **6. PDI (C)** | **0.174** | ***0.373*** | ***0.279*** | ***0.251*** | ***0.358*** | 1 |  |  |  |  |  |  |  |  |  |  |
| **7. ERI** | 0.151 | ***0.454*** | **0.178** | ***0.224*** | ***0.454*** | ***0.429*** | 1 |  |  |  |  |  |  |  |  |  |
| **8. DRSD** | 0.157 | ***0.458*** | **0.176** | 0.054 | **0.180** | ***0.235*** | ***0.309*** | 1 |  |  |  |  |  |  |  |  |
| **9. IAPSD** | 0.132 | ***0.405*** | **0.198** | 0.168 | ***0.350*** | **0.197** | ***0.274*** | ***0.533*** | 1 |  |  |  |  |  |  |  |
| **10. ILSD** | -0.043 | ***0.315*** | 0.011 | -0.056 | 0.151 | 0.154 | 0.082 | ***0.344*** | ***0.476*** | 1 |  |  |  |  |  |  |
| **11. ODSD** | 0.036 | ***0.391*** | 0.156 | 0.041 | 0.111 | **0.172** | **0.188** | ***0.739*** | ***0.518*** | ***0.324*** | 1 |  |  |  |  |  |
| **12. OVISD** | **0.178** | ***0.469*** | 0.064 | 0.043 | ***0.298*** | **0.184** | ***0.457*** | ***0.759*** | ***0.502*** | ***0.398*** | ***0.586*** | 1 |  |  |  |  |
| **13. USABP** | 0.097 | **0.181** | ***0.246*** | **0.210** | 0.069 | 0.162 | 0.058 | **0.178** | 0.107 | **-0.192** | 0.067 | 0.073 | 1 |  |  |  |
| **14. CPCCA** | ***0.224*** | **0.207** | ***0.324*** | ***0.256*** | 0.101 | **0.218** | 0.135 | ***0.228*** | **0.177** | -0.100 | 0.098 | 0.106 | ***0.804*** | 1 |  |  |
| **15. CPDW** | **0.199** | ***0.343*** | 0.151 | 0.102 | ***0.256*** | ***0.286*** | ***0.460*** | ***0.411*** | ***0.357*** | **0.189** | **0.212** | ***0.412*** | 0.012 | 0.138 | 1 |  |
| **16. CPSIMR** | 0.070 | **0.199** | 0.035 | 0.016 | 0.118 | 0.086 | ***0.280*** | ***0.267*** | 0.164 | 0.043 | 0.113 | ***0.292*** | ***0.302*** | ***0.397*** | ***0.559*** | 1 |

*Note. PDI (A/C): perception of duration of the illness (acute/ chronic), PCI**:* *perception of the consequences of the illness, PPC:* *perception of personal control, PTC**:* *perception of treatment control, CUI: coherency in understanding illness, PDI (C)**: perception of duration of the illness (cyclical), ERI:* *emotional representations related to the illness, DRSD**: disconnection/ rejection schema domain, IAPSD: impaired autonomy/ performance schema domain, ILSD: impaired limits schema domain, ODSD: other directedness schema domain, OVISD**: over-vigilance/ inhibition schema domain, USABP**: using self-active behavioral pathways to cope with pain, CPCCA: coping with pain through conscious cognitive attempts, CPDW:* *coping with pain in desperate ways, CPSIMR**:* *coping with pain by seeking ineffective medical remedies. * Table values are Spearman’s rho correlation coefficients.* ***Bold*** *indicates p < .05 and* ***italic bold*** *indicates p < .01*
